# Validation and measurement invariance of the Occupational Depression Inventory in South Africa

**DOI:** 10.1371/journal.pone.0261271

**Published:** 2021-12-16

**Authors:** Carin Hill, Leon T. de Beer, Renzo Bianchi

**Affiliations:** 1 Department of Industrial Psychology and People Management, School of Management, College of Business and Economics, University of Johannesburg, Johannesburg, South Africa; 2 WorkWell Research Unit, North-West University, Potchefstroom, South Africa; 3 Institute of Work and Organizational Psychology, University of Neuchâtel, Neuchâtel, Switzerland; Charles Sturt University - Port Macquarie Campus, AUSTRALIA

## Abstract

This study aimed to validate the recently developed Occupational Depression Inventory (ODI) in South Africa. A total of 327 employees (60% female) participated in the study. Bifactor exploratory structural equation modeling analysis indicated that the ODI can be considered essentially unidimensional. The ODI displayed strong scalability (e.g., scale-level *H* = 0.657). No monotonicity violation was detected. The reliability of the instrument, as indexed by Cronbach’s alpha, McDonald’s omega-total, Guttman’s λ_2_, and the Molenaar-Sijtsma statistic, was highly satisfactory. Measurement invariance was observed across age groups, sexes, and ethnicities, as well as between our sample and the ODI’s original validation sample. As expected, the ODI showed both a degree of convergent validity and a degree of discriminant validity vis-à-vis a measure of “cause-neutral” depressive symptoms. Moreover, the ODI manifested substantial associations, in the anticipated directions, with measures of work engagement, job satisfaction, and life satisfaction. Overall, the ODI exhibited excellent structural and psychometric properties within the South African context. Consistent with previous research, this study suggests that occupational health specialists can confidently rely on the ODI to investigate job-related distress.

## Introduction

Depression afflicts approximately 300 million people globally, and a great deal of this disease burden is in low- and middle-income countries [1]. Many of these countries are located on the African continent. There is a wide consensus on the need to optimize health research in Africa [2], in line with the sustainable development goals set by the World Health Organization [3]. The present study aimed to validate the recently developed Occupational Depression Inventory (ODI) in South Africa, which is currently considered an upper-middle-income [4], non-WEIRD (Western, Educated, Industrial, Rich, and Democratic) country.

The lifetime prevalence of depression in South Africa is close to 10% [5, 6], with a financial cost representing nearly 6% of the gross domestic product (GDP)―about US$16 billion a year. The South African Depression and Anxiety Group (SADAG) has estimated that 1 in 4 employees suffers from depression and that affected employees (a) are unable to function normally 57 days of the year and (b) take approximately 18 days off work due to depression [6, 7]. However, due to the perceived stigma associated with depression, employees are reluctant to disclose the condition as the reason for their sick leave [8]. Importantly, these estimates were pre-pandemic. The effects of the pandemic (e.g., large-scale lockdowns) on individuals already experiencing depression and borderline cases can be expected to bear on depression’s prevalence in the years to come. From an economic standpoint, pandemic-management strategies driven by governments have been accompanied by the closure of businesses and the layoff of employees. In fact, Statistics South Africa estimated in 2020 that more than 600,000 jobs were shed in the formal sector alone, and that year-on-year earnings of labor decreased [9]. In light of this state of affairs, there is little doubt that many people are exposed to additional stressors in their personal and occupational lives.

Until recently, no nosologically-grounded tool existed for assessing occupational depression, leaving the question of the prevalence of the condition unresolved. The recently developed ODI addresses this gap [10]. The ODI comprises items assessing anhedonic-somatic and dysphoric symptoms, in line with the diagnostic criteria for major depression of the *Diagnostic and Statistical Manual of Mental Disorders* (5th ed.; DSM-5) [11]. Uniquely, the items of the ODI assess these symptoms in connection to job stress. Using the ODI, practitioners and researchers can quantify depressive symptoms that individuals attribute to their work (dimensional approach) *and* establish provisional diagnoses of job-ascribed depression (categorical approach), based on an algorithm referencing DSM-5’s diagnostic criteria for major depression. The present study investigated the validity, reliability, and measurement invariance of the ODI within the South African context.

To date, the ODI has demonstrated excellent psychometric properties [10]. Results from the seminal ODI article showed that the ODI has an essentially unidimensional structure. Such findings were obtained based on exploratory structural equation modeling (ESEM) bifactor analysis. The alpha and omega reliabilities of the ODI proved highly satisfactory. The ODI exhibited both a degree of convergent validity and a degree of discriminant validity vis-à-vis measures of “cause-neutral” depressive symptoms (i.e., depression scales that do not ask respondents to make attributions about the causes of their symptoms), namely, the 10-item version of the Center for Epidemiologic Studies-Depression scale (CES-D) and the Depression subscale of the Hospital Anxiety and Depression Scale (HADS-D). The ODI was found to correlate substantially with measures of work engagement (specifically, dedication to work), job satisfaction, and life satisfaction, among others.

In the current study, we investigated how the ODI behaved in the South African context focusing on core psychometric properties of the instrument, including factorial validity, convergent validity, and discriminant validity. We examined the relationships between the ODI and measures of cause-neutral depressive symptoms, life satisfaction, job satisfaction, and work engagement. Work engagement is described as a positive, work-related state of mind comprising vigor, dedication, and absorption [12]. Work engagement―especially its vigor component―has shown negative associations with depression over time [13]. Bianchi and Schonfeld [10] found dedication to work to correlate negatively with occupational depression. The present study also considered job satisfaction and life satisfaction―two variables that correlated negatively with occupational depression in previous research [10]. Because the ODI focuses on depressive symptoms that individuals attribute to their work, the ODI can be expected to (a) correlate negatively with both job satisfaction and life satisfaction and (b) correlate more strongly with job satisfaction than with life satisfaction. On a more general note, there is evidence for negative associations between cause-neutral measures of depression and measures of job satisfaction (e.g., [14]) and life satisfaction (e.g., [15]).

Given the use and abuse of psychometrics in South Africa during the Apartheid era, legislations were crafted to ensure that users of psychological tests can present clear evidence of the reliability, validity, and between-group (e.g., cross-ethnicity) equivalence of their measures [16, 17]. Specifically, psychological tests are required to be “fair”; they should not be prejudicial to any particular group. Prejudice can manifest itself, for instance, when comparing metrics on tests without support for between-group equivalence. On a different note, it should be underlined that the psychological literature has been dominated by “WEIRD studies,” pointing to a need for more research within non-WEIRD contexts such as the South African context [18]. In an effort to take these various issues into account, the current study implemented measurement invariance analyses of the ODI. More specifically, we examined whether the ODI behaved in an invariant manner (a) across the age groups, sexes, and ethnicities involved in the present study and (b) in the present study by comparison with the original ODI study conducted by Bianchi and Schonfeld [10]. Measurement invariance across these various (sub)samples would not only suggest that the ODI is a valid, reliable, and fair tool in the South African context; it would also demonstrate the potential of the instrument for international comparative studies.

Based on the state of the art reviewed above, we formulated and tested the following hypotheses:

H1: The ODI has an essentially unidimensional structure.
H2: The ODI shows measurement invariance across age groups, sexes, and ethnicities.
H3: The ODI shows measurement invariance when comparing the South African sample to an international, WEIRD sample.
H4: The ODI shows both a degree of convergent validity and a degree of discriminant validity with a measure of cause-neutral depressive symptoms.
H5: The ODI is negatively related to work engagement, job satisfaction, and life satisfaction.

## Materials and methods

### Study sample

The study received ethics clearance from the University of MASKED FOR REVIEW (IPPM-2020-464). A purposive sampling strategy was used to collect the data. To be eligible to participate in the study, an individual had to be (a) at least 18 years of age, (b) a South African citizen currently working within the country, and (c) employed in the formal sector [9]. The study was advertised on different social media platforms (e.g., Facebook and LinkedIn) with a hyperlink to an electronic questionnaire. Information required for consent was presented first and accepted by potential respondents before they could complete the online survey.

A total of 327 individuals took part in the study. Age was divided into five categories: 18–25 years old (category 1; 24%), 26–37 years old (category 2; 45%), 38–45 years old (category 3; 16%), 46–60 years old (category 4; 11.00%), and > 60 years old (category 5; 4%). Of the 327 participants, 130 indicated to be men (39.80%) and 196 to be women (59.90%); one participant did not respond. Finally, in line with the designations used by the Employee Equity Act [19], the study sample included 124 African employees (38%), 51 Indian employees (16%), 12 Colored employees (an official term designating people with a mixed ethnic origin; 4%), 135 White employees (41%), and 4 Asian employees (1%). One participant did not respond to the ethnicity item.

### Measures of interest

ODI. As noted earlier, the ODI was developed with reference to the diagnostic criteria for major depressive disorder found in the DSM-5 [11]. The ODI thus covers anhedonia, depressed mood, sleep alterations, fatigue/loss of energy, appetite alterations, feelings of worthlessness, cognitive impairment, psychomotor alterations, and suicidal ideation. Here are two sample items: “My experience at work made me feel like a failure”; “I felt exhausted because of my work.” The complete item list is available in Bianchi and Schonfeld’s [10] article. Consistent with DSM-5 diagnostic criteria, the symptoms are assessed within a two-week time window. Respondents use a 4-point frequency scale (from 0 for “never or almost never” to 3 for “nearly every day”) to report on their symptoms. The instrument includes a subsidiary question about turnover intention, offering three response options (“yes,” “no,” and “I don’t know”).

The ODI allows investigators to examine work-attributed depressive symptoms from a dimensional (continuum-based) and a categorical (or diagnostic) standpoint. On the one hand, investigators assess the severity of work-attributed depressive symptoms. On the other hand, investigators are provided with an algorithm for establishing provisional diagnoses of job-ascribed depression [10]. This dual-lens approach is in keeping with recent developments in psychopathological science [20–22]. The ODI can be used free of charge. The ODI is currently available in English, French, and Spanish. This study employed the English version of the instrument because English is the generally accepted business language among South African employees. Of our participants showing no missing responses on the ODI (*n* = 324), about 7% (*n* = 24) were identified as likely cases of job-ascribed depression. Of our participants showing no missing responses on the turnover intention item of the ODI (*n* = 326), about 39% (*n* = 127) indicated that they were considering leaving their current job or position due to job-related distress.

“Cause-Neutral” depressive symptoms. Cause-neutral depressive symptoms were assessed with the Depression subscale of the Depression Anxiety Stress Scales-21 (DASS-21-D; [23, 24]). In the DASS-21-D, depressive symptoms are assessed over a one-week period using a 4-point rating scale (from 1 for “Did not apply to me at all” to 4 for “Applied to me very much or most of the time”). A sample item is: “I felt that life was meaningless.” Cronbach’s alpha (α) and McDonald’s omega-total (ω_t_) were both 0.931.

Work engagement. Work engagement was assessed with the ultra-short, 3-item version of the Utrecht Work Engagement Scale (UWES-3; [25]). Participants responded using a rating scale from 1 for “Never” to 7 for “Always.” A sample item is: “I am enthusiastic about my job.” Cronbach’s α was 0.870 and McDonald’s ω_t_, 0.876.

Job satisfaction and life satisfaction. We assessed job satisfaction and life satisfaction using one-item measures rated on a 7-point scale (from 1 for “Extremely dissatisfied” to 7 for “Extremely satisfied”). The items were: “Overall, I am satisfied with my job”; “Overall, I am satisfied with my life.” There is robust evidence that job satisfaction and life satisfaction can be effectively measured using one-item measures [26, 27].

### Data analyses

Analyses of the ODI. We examined the factorial structure of the ODI based on bifactor ESEM analysis in Mplus 8.6 [28]. We treated the items as ordinal and used the weighted least squares—mean and variance adjusted—(WLSMV) estimator, which robustly deals with potentially non-normal data [29, 30]. Because our approach was primarily confirmatory, we relied on a target rotation (TR). An advantage of the TR is that nontarget loadings are *not* fixed to be equal to 0. Instead, nontarget loadings are “encouraged” to get as close to 0 as possible. Bifactor ESEM analysis with a TR departs from the somewhat unrealistic assumptions attached to the Independent Cluster Model underlying common-practice confirmatory factor analysis [31]. As per Bianchi and Schonfeld [10], we considered two specific factors in addition to the general factor. The first specific factor targeted the “anhedonic-somatic” items of the ODI (Items 1, 3, 4, 5, 7, and 8). The second specific factor targeted the “dysphoric” items of the ODI (Items 2, 6, and 9). We computed the Explained Common Variance (ECV) statistic to estimate the importance of the general factor in accounting for the common variance extracted [32, 33]. An ECV index exceeding 0.80 is suggestive of essential unidimensionality. In addition, we investigated the scalability (homogeneity) and monotonicity properties of the ODI using the Mokken package version 3.0.3 [34] in R version 4.0.3 [35]. Scalability and monotonicity are two core components of Mokken scale analysis, a method anchored in nonparametric Item Response Theory. Scalability was estimated based on Loevinger’s *H* coefficient, considered at the level of items, item pairs, and the entire scale. Item-level *H* coefficients should be > 0.30. Pairwise *H* coefficients should be > 0.00. A scale is considered weak if 0.30 ≤ *H* < 0.40; moderate, if 0.40 ≤ *H* < 0.50; and strong, if *H* ≥ 0.50. The predicates “weak,” “moderate,” and “strong” characterize the extent to which the ordering of individuals by test score reflects their ordering on the latent variable. We also explored the ODI’s scalability using the Automated Item Selection Procedure (AISP), which employs user-defined thresholds of homogeneity based on the scale-level *H* coefficient. As recommended (e.g., [36]), we explored thresholds ranging from 0.30 to 0.55 in increments of 0.05. We inquired into monotonicity violations in terms of their presence, statistical significance, and seriousness—as indexed by the *crit* statistic [36]. We scrutinized the reliability of the ODI based Cronbach’s α, McDonald’s ω_t_, Guttman’s λ2, and the Molenaar-Sijtsma (MS) statistic.

We examined measurement invariance across age groups, sexes, and ethnicities, as well as between our sample and the ODI’s original validation sample, which involved 2,254 participants [10]. We focused on configural, metric, and scalar invariance [37]. Configural invariance deals with the issue of whether the overall factorial structure fits well in all groups of interest. Metric invariance addresses the issue of whether factor loadings can be regarded as equivalent across the groups. Scalar invariance concerns the issue of whether item thresholds (when examining ordinal data) are equivalent across groups. We relied on commonly accepted rules of thumb, i.e., between-model maximum delta change (Δ) of -0.010 for CFI and 0.015 for RMSEA [38, 39]. Additionally, we examined ΔSRMR with a threshold of 0.015, as was the case with ΔRMSEA. Recent research suggests that SRMR is a more relevant index than RMSEA with ordinal data [40], as well as with models exhibiting small degrees of freedom [41].

Finally, we used the lordif package version 0.3–3 [42] in R version 4.0.3 [35] to test for differential item functioning (DIF) across age groups, sexes, ethnicities, and samples. Using the lordif package, different model types are compared (see [42] for a detailed description of the analysis). Uniform DIF is identified when there is a significant chi-square test value (*p* < 0.01) between models 1 (M1) and 2 (M2), and non-uniform bias is identified when there is a significant chi-square test value (*p* < 0.01) between models 2 (M2) and 3 (M3). We considered DIF for an item to be likely when there was evidence of either a uniform DIF or a non-uniform bias. However, to consider the impact of any potential DIF, we reported on chi-square difference testing for the different models and the McFadden pseudo-R^2^ and paid specific attention to an upper cut-off of 5% for the change in the beta parameter between the models [42].

Analyses of the ODI in relation to the DASS-21-D. We investigated the convergent validity and discriminant validity of the ODI vis-à-vis the DASS-21-D within an ESEM bifactor analytic framework. As was previously the case, we treated the items as ordinal, used the WLSMV estimator, and relied on a TR. We considered two bifactors in addition to the general factor—a first bifactor for the items of the ODI and a second bifactor for the items of the DASS-21-D. We examined the ECV index to identify potential deviations from essential unidimensionality. In addition, we inspected Pearson and Spearman correlations between the ODI and the DASS-21-D.

Analyses of the ODI in relation to work, non-work, and socio-demographic variables. We examined whether and how the ODI related to work engagement, job satisfaction, life satisfaction, sex, and age using both Pearson and Spearman correlations.

## Results

### Dimensionality and reliability

**Fig 1:**
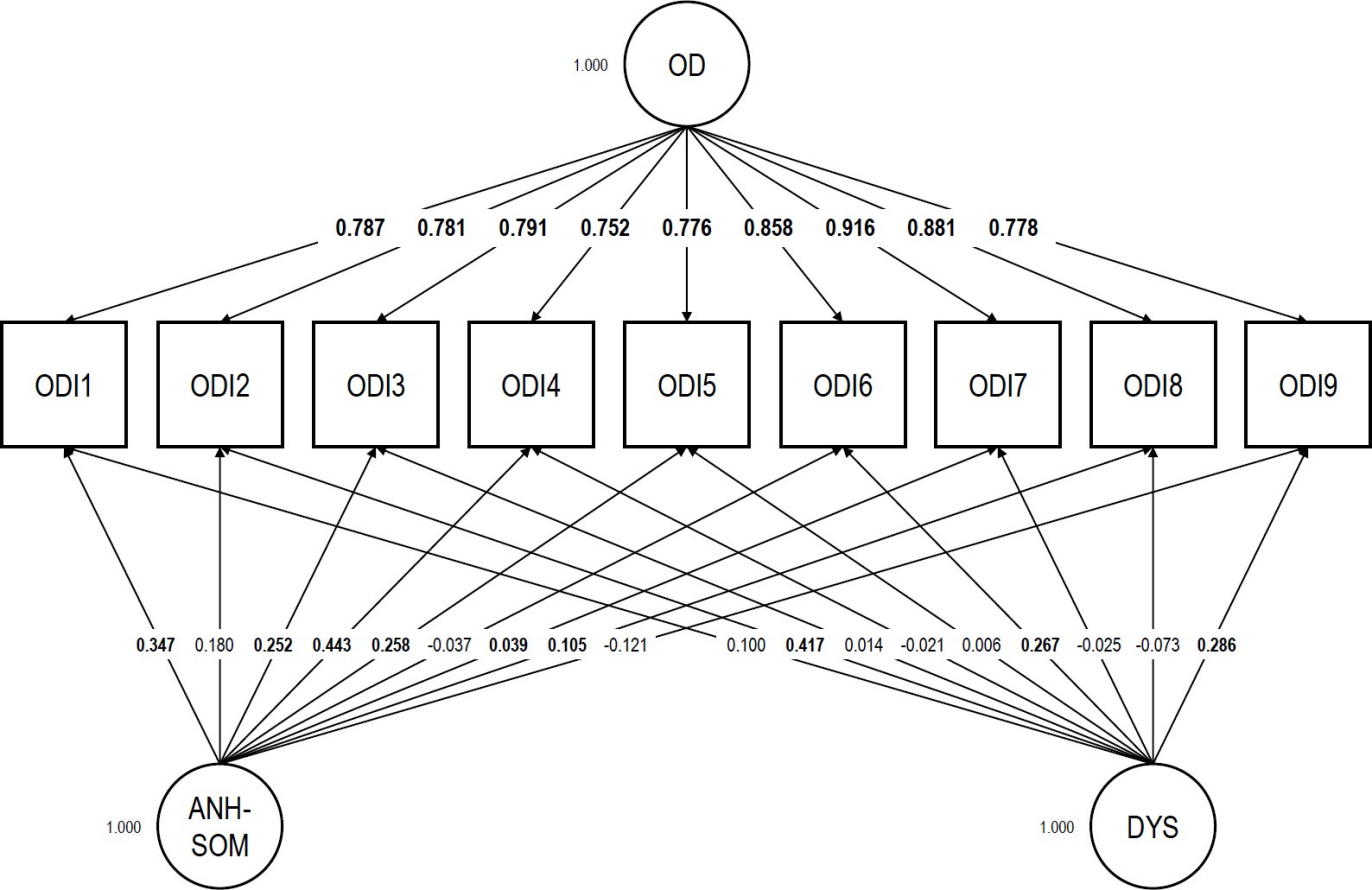
Exploratory structural equation modeling bifactor analytic model of the Occupational Depression Inventory―factor loadings. Target loadings are bolded. RMSEA = 0.019; CFI = 1.000; TLI = 0.999; SRMR = 0.010; *χ^2^* (12) = 13.397. OD: general Occupational Depression factor; ANH-SOM: Anhedonic-Somatic bifactor; DYS: Dysphoric bifactor.

Our bifactor ESEM analytic model of the ODI showed a satisfactory fit: RMSEA = 0.019; CFI = 1.000; TLI = 0.999; SRMR = 0.010; *χ^2^* (12) = 13.397. The factor loadings are displayed in Fig 1. All items loaded strongly on the general factor (from 0.752 to 0.916; *M* = 0.813, *SD* = 0.057) and more strongly on the general factor than on any of the specific factors. The specific factors were weak but did not entirely collapse. The general factor accounted for about 88% of the common variance extracted, a proportion indicative of essential unidimensionality [32, 33]. The subscale-level ECV index had a value of 0.892 for “anhedonic-somatic” items and 0.840 for “dysphoric” items, pointing to homogeneous contributions of both item subsets to the scale-level ECV index. Unidimensionality was also reflected in the outcomes of our scalability analysis. Item-level *H* coefficients ranged from 0.613 to 0.692 (*SE*s ranging from 0.026 to 0.043) and the scale-level *H* coefficient was 0.657 (*SE* = 0.024). No pairwise *H* coefficient was problematic. The AISP identified only one scale comprising all ODI items at every threshold tested, including the most stringent one (0.55). No item was thus found to be unscalable. No monotonicity violation was detected (see S1 File). The ODI exhibited strong reliability: Cronbach’s α was 0.926; McDonald’s ω_t_, 0.928; Guttman’s λ_2_, 0.928; and the MS statistic, 0.931. These results support H1.

### Measurement invariance and DIF

For the measurement invariance analyses, a unidimensional model was used. The unidimensional model for the overall sample exhibited a satisfactory fit: RMSEA = 0.079; CFI = 0.991; TLI = 0.988; SRMR = 0.029; *χ^2^* (27) = 81.732. Factor loadings were all strong, ranging from 0.771 to 0.896 (*M* = 0.837, *SD* = 0.037). The invariance tests for the unidimensional specification showed strong invariance across all groups and model conditions under scrutiny (Table 1). Specifically, CFI values never decreased by more than 0.002, a trend that is indicative of measurement invariance even when evaluated against highly conservative cut-points [43]. In parallel, RMSEA values never increased by more than 0.004, and ΔSRMR ranged from 0.000 to 0.004, again indicating that measurement invariance applied. Interestingly, RMSEA often showed superiority in more constrained models. Overall, we found evidence that the ODI behaves similarly across groups, allowing for meaningful comparisons between groups. These results support H2 and H3.

**Table 1:** Summary of measurement invariance analysis.

**Age groups**								
**Model**	***χ*** ^***2***^	**d*f***	**CFI**	**ΔCFI**	**RMSEA**	**ΔRMSEA**	**SRMR**	**ΔSRMR**
Configural	157.17	108	0.994	―	0.076	―	0.045	―
Metric	180.96	120	0.994	0.000	0.069	-0.007	0.048	0.003
Scalar	224.97	183	0.995	0.001	0.051	-0.018	0.051	0.003
**Sexes**								
**Model**	***χ*** ^***2***^	**d*f***	**CFI**	**ΔCFI**	**RMSEA**	**ΔRMSEA**	**SRMR**	**ΔSRMR**
Configural	99.69	54	0.993	―	0.072	―	0.032	―
Metric	121.03	62	0.991	-0.002	0.076	0.004	0.036	0.004
Scalar	147.30	79	0.989	-0.002	0.073	-0.003	0.038	0.002
**Ethnicities**								
**Model**	***χ*** ^***2***^	**d*f***	**CFI**	**ΔCFI**	**RMSEA**	**ΔRMSEA**	**SRMR**	**ΔSRMR**
Configural	146.11	81	0.991	―	0.088	―	0.042	―
Metric	162.14	97	0.991	0.000	0.081	-0.007	0.044	0.002
Scalar	191.64	131	0.992	0.001	0.067	-0.014	0.047	0.003
**Samples**								
**Model**	***χ*** ^***2***^	**d*f***	**CFI**	**ΔCFI**	**RMSEA**	**ΔRMSEA**	**SRMR**	**ΔSRMR**
Configural	675.08	54	0.987	―	0.094	―	0.033	―
Metric	681.14	62	0.987	0.000	0.088	-0.006	0.033	0.000
Scalar	711.92	79	0.987	0.000	0.079	-0.009	0.035	0.002

Notes. *χ*^*2*^ = chi-square; d*f* = degrees of freedom; CFI = comparative fit index; ΔCFI = delta (change in) CFI; RMSEA = root mean square error of approximation; ΔRMSEA = delta (change in) RMSEA; SRMR = standardized root mean residual; ΔSRMR = delta (change in) SRMR.

The results from the DIF tests were consistent with the results of the abovementioned measurement invariance analyses. Some of the items were flagged for statistical significance, but the impact of DIF for these items was of negligible magnitude (S2 File). All in all, the ODI thus presented homogeneous properties across the various groups of interest.

### Convergent and discriminant validity

In our second bifactor ESEM model, we found the ODI to exhibit both a degree of convergent validity and a degree of discriminant validity vis-à-vis the DASS-21-D (Table 2). This bifactor ESEM model also had a satisfactory fit: RMSEA = 0.040; CFI = 0.996; TLI = 0.994; SRMR = 0.018; *χ^2^* (75) = 115.128. On the one hand, all ODI and DASS-21-D items loaded substantially on the general factor (*M* = 0.742, *SD* = 0.136), a finding suggestive of a degree of convergent validity. The mean factor loading on the general factor was 0.864 for DASS-21-D items (*SD* = 0.045) and 0.647 for ODI items (*SD* = 0.099). On the other hand, the ODI bifactor was relatively strong, and the scale-level ECV index linked to ODI items had a value of only 0.583, suggesting a degree of discriminant validity. The picture emerging from our ESEM bifactor analysis was consistent with the correlations that we observed between the ODI and the DASS-21-D: *r* = 0.697; *ρ* = 0.671. These results support H4.

**Table 2:** Bifactor exploratory structural equation modeling analysis of the Occupational Depression Inventory (ODI) and the Depression subscale of the Depression Anxiety Stress Scales-21 (DASS-21-D).

Item	GF	SF1	SF2	C	I-ECV	S-ECV	ECV
**DASS-21-D Item 1**	0.812	0.256	0.109	0.736	0.896	0.909	0.726
**DASS-21-D Item 2**	0.798	0.301^a^	0.070	0.733	0.869		
**DASS-21-D Item 3**	0.891	0.316^a^	-0.033	0.895	0.887		
**DASS-21-D Item 4**	0.864	0.252	0.046	0.812	0.919		
**DASS-21-D Item 5**	0.888	0.279	0.028	0.868	0.908		
**DASS-21-D Item 6**	0.925	-0.164	-0.144	0.903	0.948		
**DASS-21-D Item 7**	0.872	-0.203	-0.087	0.810	0.939		
**ODI Item 1**	0.551	0.161	0.657^a^	0.762	0.398	0.583	
**ODI Item 2**	0.730	0.095	0.427^a^	0.724	0.736		
**ODI Item 3**	0.577	-0.018	0.594^a^	0.686	0.485		
**ODI Item 4**	0.509	0.140	0.661^a^	0.716	0.362		
**ODI Item 5**	0.607	-0.024	0.544^a^	0.665	0.554		
**ODI Item 6**	0.763	-0.066	0.422^a^	0.764	0.762		
**ODI Item 7**	0.661	-0.090	0.609^a^	0.816	0.535		
**ODI Item 8**	0.629	-0.030	0.607^a^	0.765	0.517		
**ODI Item 9**	0.796	-0.099	0.251	0.706	0.897		

Notes. RMSEA = 0.040; CFI = 0.996; TLI = 0.994; SRMR = 0.018; χ^2^ (75) = 115.128. GF: general factor; SF1: specific factor with DASS-21-D items as targets; SF2: specific factor with ODI items as targets; C: communality; ECV: Explained Common Variance; S-ECV: scale-level ECV; I-ECV: item-level ECV.

^a^ Bifactor loadings ≥ 0.300.

### The ODI in relation to work, non-work, and socio-demographic variables

The ODI correlated with our other variables of interest in the expected directions (Table 3). Moderate to large negative correlations were observed with work engagement (*r* = -0.465, *ρ* = -0.458), job satisfaction (*r* = -0.566, *ρ* = *-*0.568), and life satisfaction (*r* = -0.430, *ρ* = -0.382). Work engagement correlated positively with job satisfaction (*r* = 0.694, *ρ* = 0.699; large association) and life satisfaction (*r* = 0.473, *ρ* = 0.473; moderate association). The correlation between job and life satisfaction was positive and large (*r* = 0.594, *ρ* = 0.600). Finally, the correlations involving sex and age were small in magnitude and statistically nonsignificant. These results support H5.

**Table 3:** Pearson and Spearman correlations among the main study variables.

	*M*	*SD*	Min.	Max.	1.	2.	3.	4.	5.	6.	7.
**1. ODI (0–3)**	0.886	0.775	0.000	3.000	―	0.671	-0.458	-0.568	-0.382	-0.072	0.039
**2. DASS-21-D (1–4)**	1.707	0.713	1.000	4.000	0.697	―	-0.524	-0.574	-0.555	-0.065	-0.056
**3. Work engagement (1–7)**	4.598	1.289	1.000	7.000	-0.465	-0.534	―	0.699	0.473	0.037	0.067
**4. Job satisfaction (1–7)**	4.508	1.636	1	7	-0.566	-0.581	0.694	―	0.600	0.014	-0.037
**5. Life satisfaction (1–7)**	4.993	1.346	1	7	-0.430	-0.559	0.473	0.594	―	0.065	-0.044
**6. Sex (0/1)**	0.399	0.490	―	―	-0.067	-0.031	0.026	0.008	0.034	―	-0.085
**7. Age (categories)**	―	―	1	5	0.033	-0.041	0.092	0.005	-0.071	-0.085	―

Notes. Pearson correlations are displayed below the diagonal; Spearman correlations are displayed above the diagonal. All correlations are statistically significant at p < 0.001, except the correlations involving sex and age, of which none are statistically significant (all *ps* > .05). ODI: Occupational Depression Inventory; DASS-21-D: Depression subscale of the Depression Anxiety Stress Scales-21; M: mean; SD: standard deviation. Sex was coded “0” for female and “1” for male.

## Discussion

This study aimed to validate the ODI, a recently developed measure of work-attributed depressive symptoms, in South Africa. We investigated the structural and psychometric properties of the ODI based on advanced statistical analyses, including bifactor ESEM analysis [31–33]. The study’s results indicate that the ODI behaves very satisfactorily within the South African context. Our results are highly consistent with those obtained in the ODI’s initial validation study [10].

In terms of factorial structure, the ODI met the requirements for essential unidimensionality. Our bifactor ESEM analysis revealed a robust general factor, accounting for a critical proportion of the common variance extracted—88%. These results dovetail with those of Bianchi and Schonfeld [10]. Indeed, in these authors’ study, the general factor explained 89% of the common variance extracted. Investigating measurement invariance for a one-dimensional ODI model, we found measurement invariance to hold across sexes, age groups, and ethnicities, as well as between our sample and the ODI’s original validation sample examined by Bianchi and Schonfeld [10]. Such results suggest that the ODI had essentially the same meaning for the various groups under scrutiny. We note that our study is the first to investigate measurement invariance in the ODI. Our scalability analysis was consistent with the observation that ODI items capture a unified phenomenon. In addition, we did not detect any violation of monotonicity—suggesting that respondents can be ordered on the latent continuum based on their total score—and found the ODI to display high total-score reliability. These results are, again, consistent with Bianchi and Schonfeld’s [10].

As hypothesized, the ODI showed both a degree of convergent validity and a degree of discriminant validity vis-à-vis a cause-neutral measure of depressive symptoms—the DASS-21-D. Similar results were obtained in the ODI’s original validation study, in which Bianchi and Schonfeld [10] assessed cause-neutral depressive symptoms with the CES-D and the HADS-D. Our study thus documents the expected combination of convergent validity and discriminant validity using yet another cause-neutral depression scale. We underline that such a combination was anticipated because, at a population level, all patients experiencing a job-ascribed depression are expected to be identified as depressed in a cause-neutral assessment of clinical depression whereas only some of the patients identified as depressed in a cause-neutral assessment of clinical depression are expected to experience a job-ascribed depression. Put differently, a degree of convergent validity should manifest itself because both the ODI and cause-neutral depression scales deal with depressive symptoms, and a degree of discriminant validity is likely because, by contrast with cause-neutral measures of depression, the ODI assesses work-attributed symptoms.

As anticipated, we found the ODI to exhibit substantial negative associations with work engagement, job satisfaction, and life satisfaction. These results are in keeping with those obtained in research on both depression and job-related distress [13, 15, 44–46]. Our findings are also consistent with those of Bianchi and Schonfeld’s [10] ODI study. As was the case in these authors’ study, we found the ODI to correlate more strongly with our measure of job satisfaction than with our measure of life satisfaction. The links that we observed between the ODI and work engagement, job satisfaction, and life satisfaction inform the ODI’s nomological network and support the ODI’s criterion validity.

### Limitations

Our study is not without limitations. First, we relied on a non-probability sampling method. As a result, it is unclear how representative our study sample is of its reference population and result generalization should be considered with caution. We note that gathering a representative sample of the South African working population would have been extremely challenging given the pandemic context. Second, although cross-sectional data are appropriate for a validation study that is not focused on causality issues, such as ours, longitudinal data may have provided insights into the impact of occupational depression on work and non-work factors over time [47]. Furthermore, measurement invariance across time could have been examined. Third, age was assessed categorically. We underline, however, that our approach to age assessment was consistent with the South Africa’s Protection of Personal Information Act.

### Conclusion

We found the ODI to have excellent structural and psychometric properties and to behave as satisfactorily within the South African context as within the U.S., New Zealand, and French contexts [10]. Given the growing concerns surrounding job-related distress and sick leave for psychological reasons, the development of valid and reliable assessment tools is crucial. Without such tools, our ability to identify individuals needing help or deal with depressogenic working conditions within organizations (e.g., harmful management styles) is likely to be impeded. This study suggests that the ODI can be a useful instrument in the hands of South African occupational health specialists.

As we close this paper, we would like to underline that the etiology of (occupational) depression is best understood through the dynamic interplay between the individual, i.e., internal dispositions, and the individual’s environment, i.e., external conditions [48–52]. On this basis, it would be unwise to neglect or downplay the role of job characteristics and working conditions (e.g., management styles involving double-binds) when investigating the issue of workplace depression. To deal effectively with job-related distress (e.g., in terms of interventions and public health policies), the importance of a balanced and comprehensive etiological approach cannot be overstated. Notably, individual and organizational factors need to be examined in a coordinated manner [53].

Future ODI research could benefit from a combination of subjective and objective (i.e., non-self-reported) indicators (e.g., to assess job performance, working conditions, or workers’ health status). A couple of ODI studies recently adopted such an approach in investigating the link between occupational depression and cognitive performance [54, 55]. In addition, implementing multi-wave designs would be important in order to address effect directionality issues and better identify predictors and outcomes of occupational depression.

## Supporting information

S1 File (PDF)

S2 File (PDF)
