# Supplementary figures and images for: Validation and measurement invariance of the Occupational Depression Inventory in South Africa
Source: PLoS One. 2021 Dec 16;16(12):e0261271. doi: 10.1371/journal.pone.0261271 (PMC8675679; doi:10.1371/journal.pone.0261271)

**odi1**

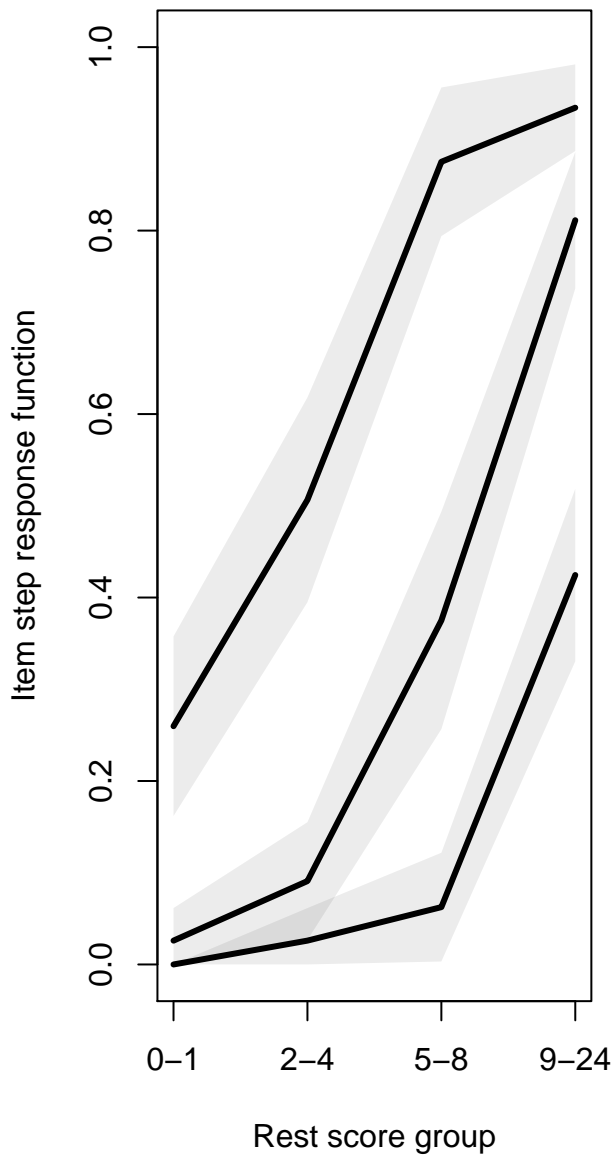

**odi1**

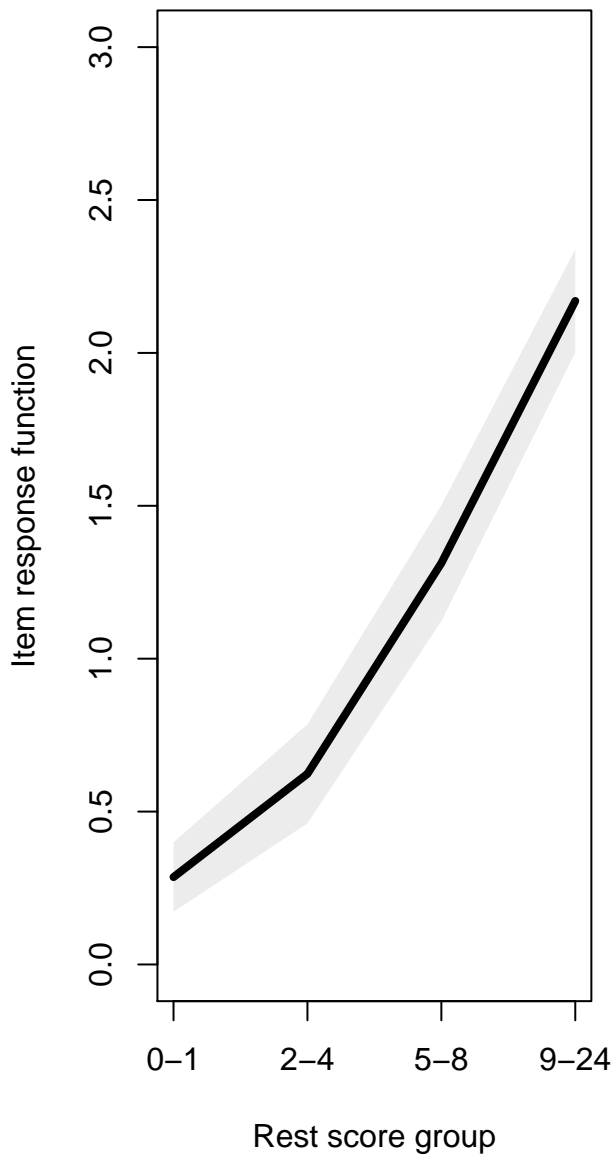

**odi2**

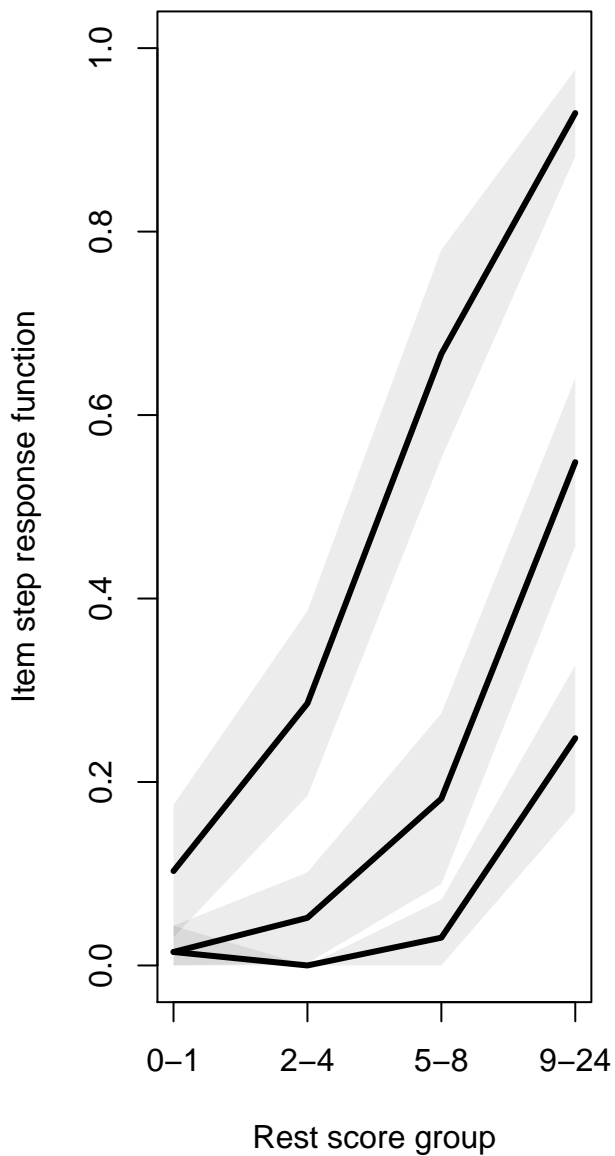

**odi2**

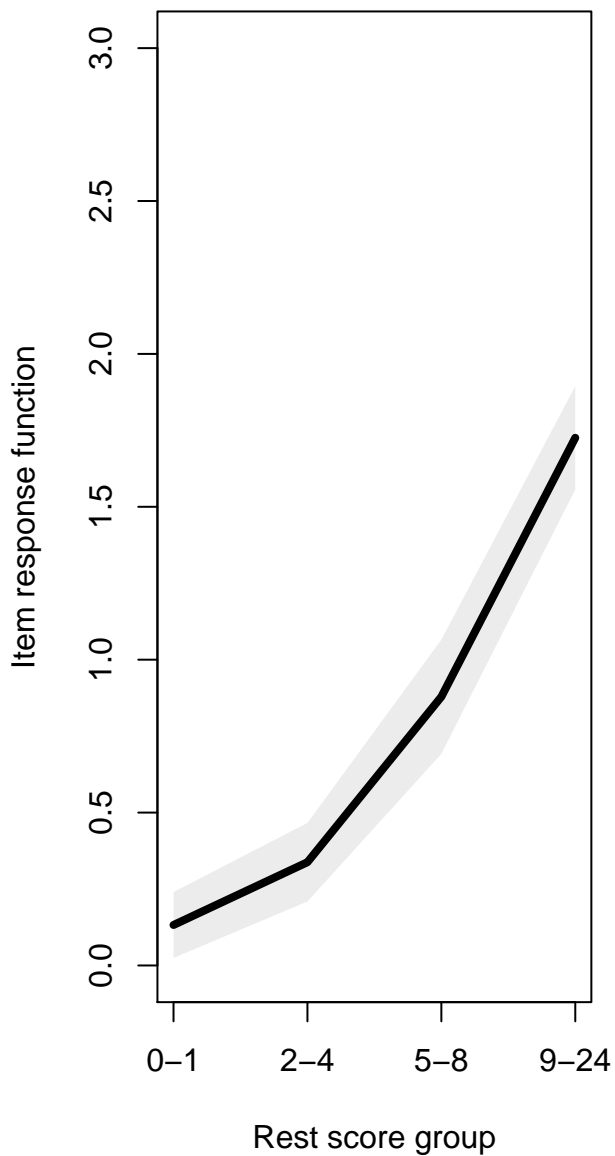

**odi3**

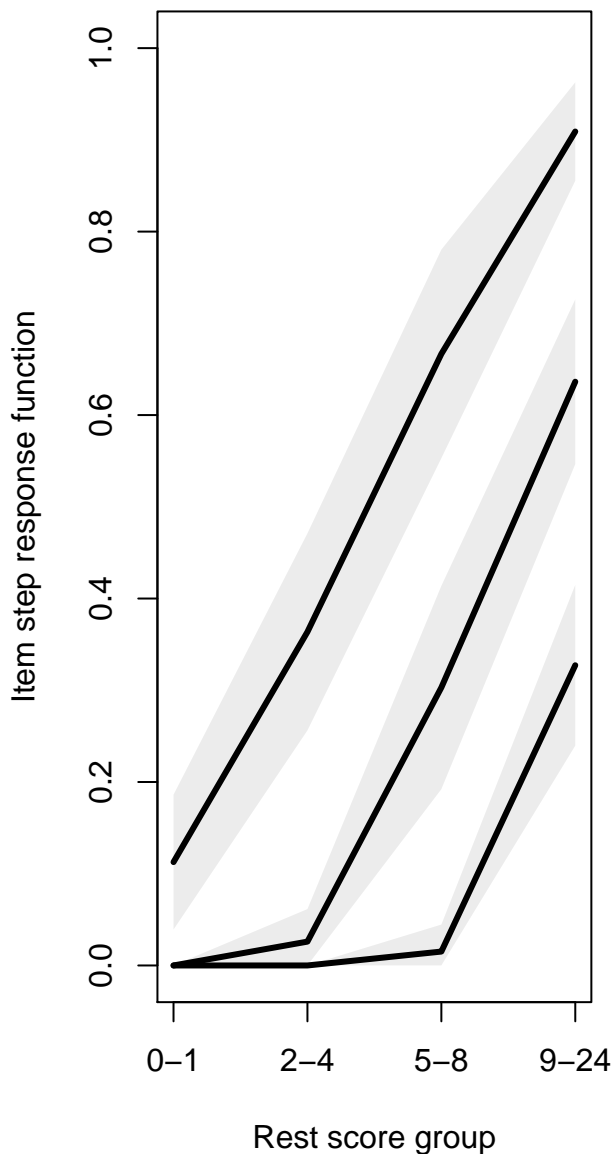

**odi3**

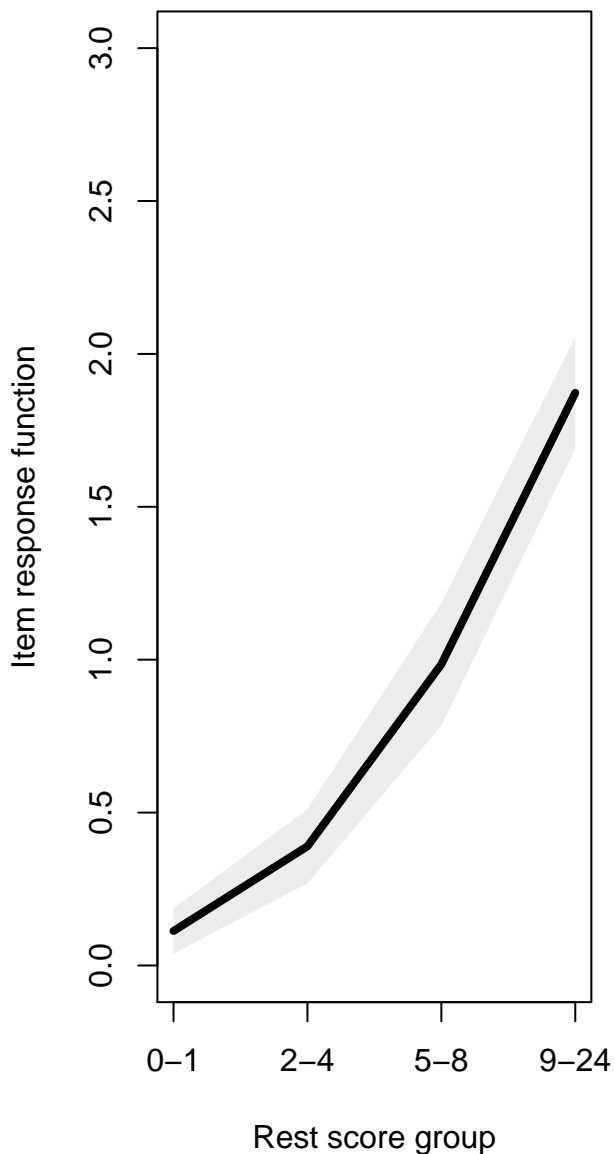

**odi4**

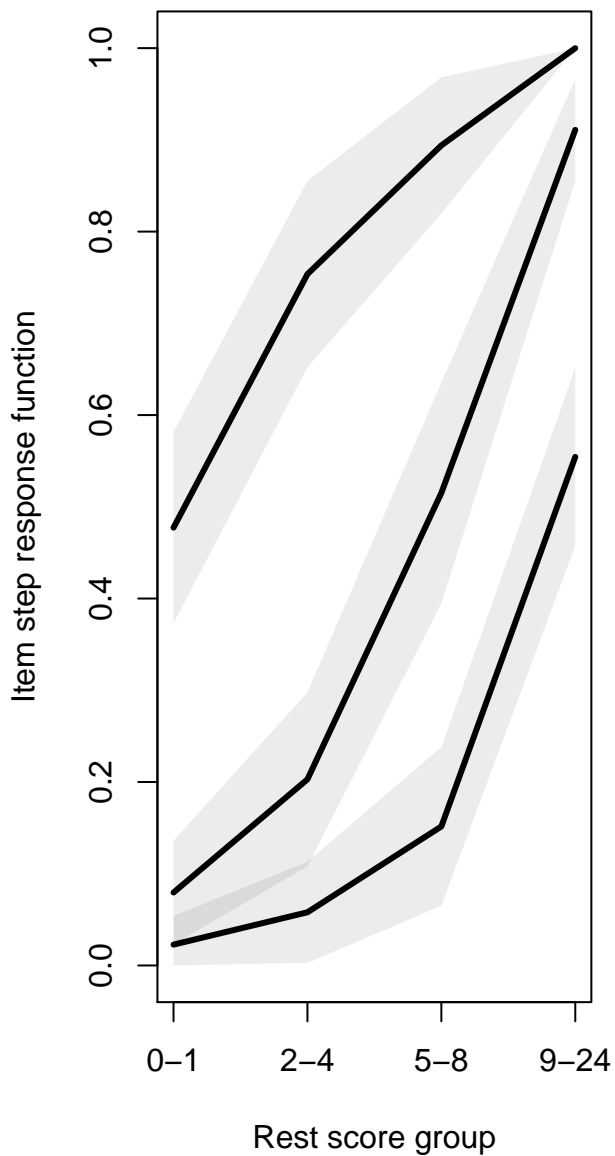

**odi4**

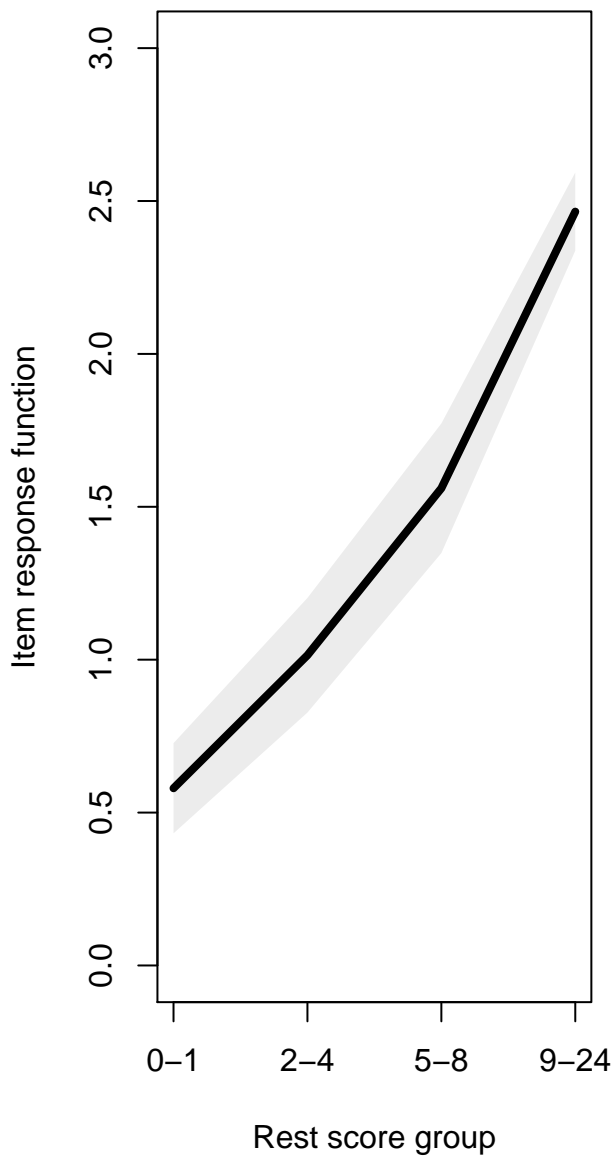

**odi5**

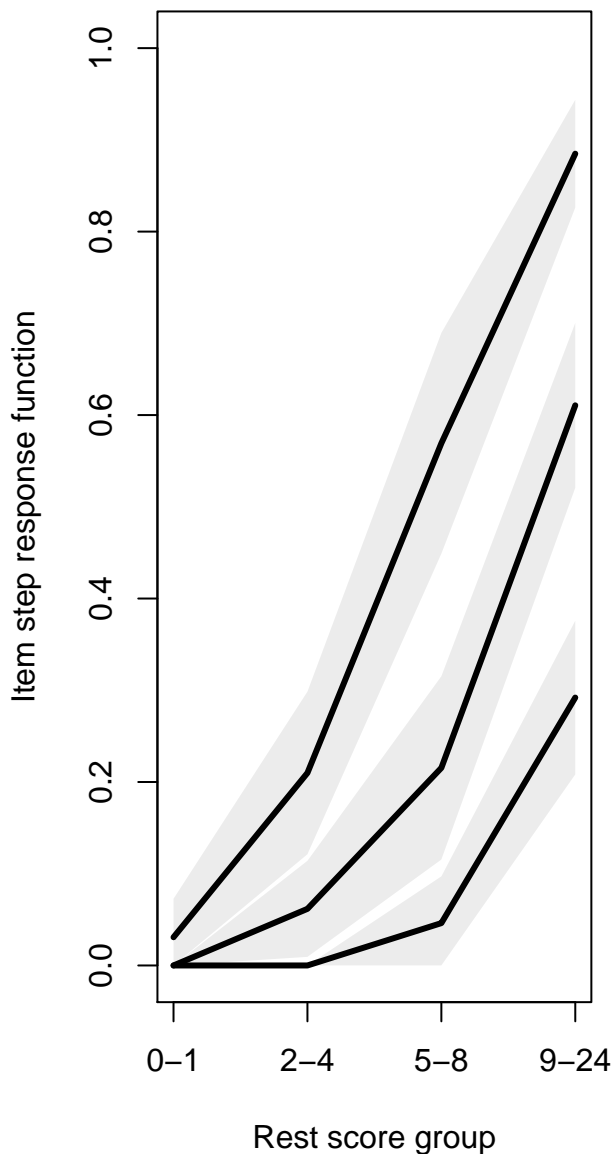

**odi5**

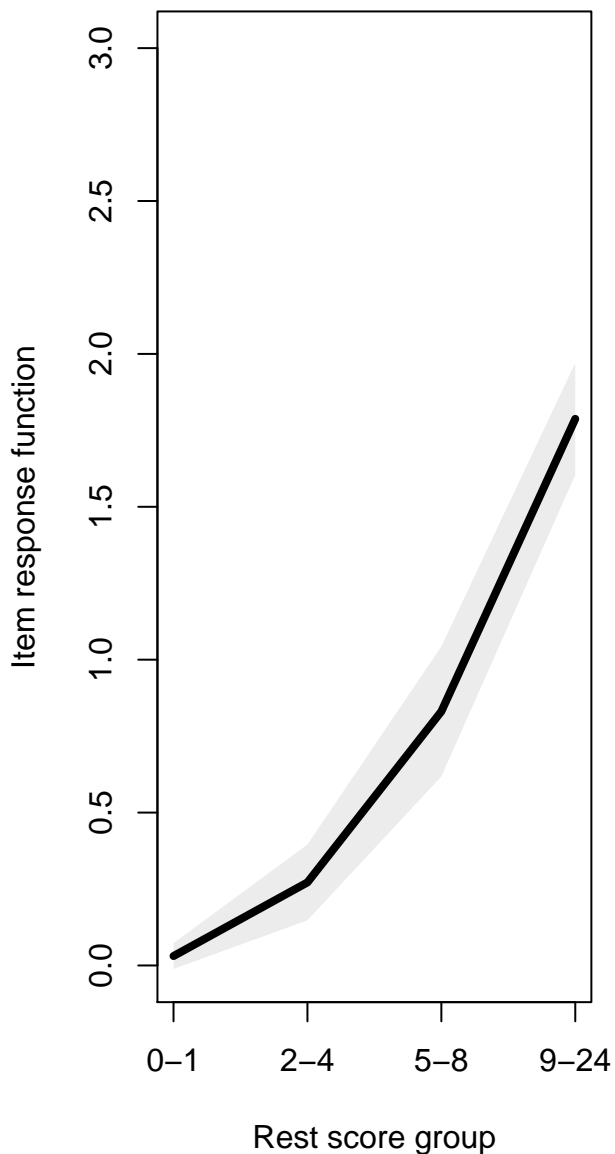

**odi6**

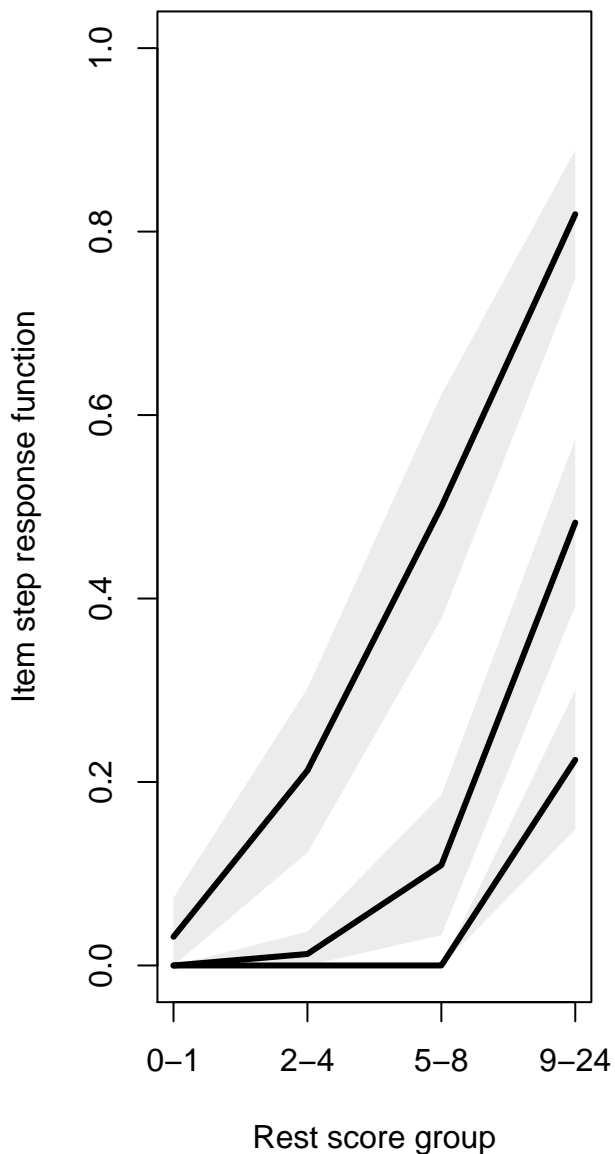

**odi6**

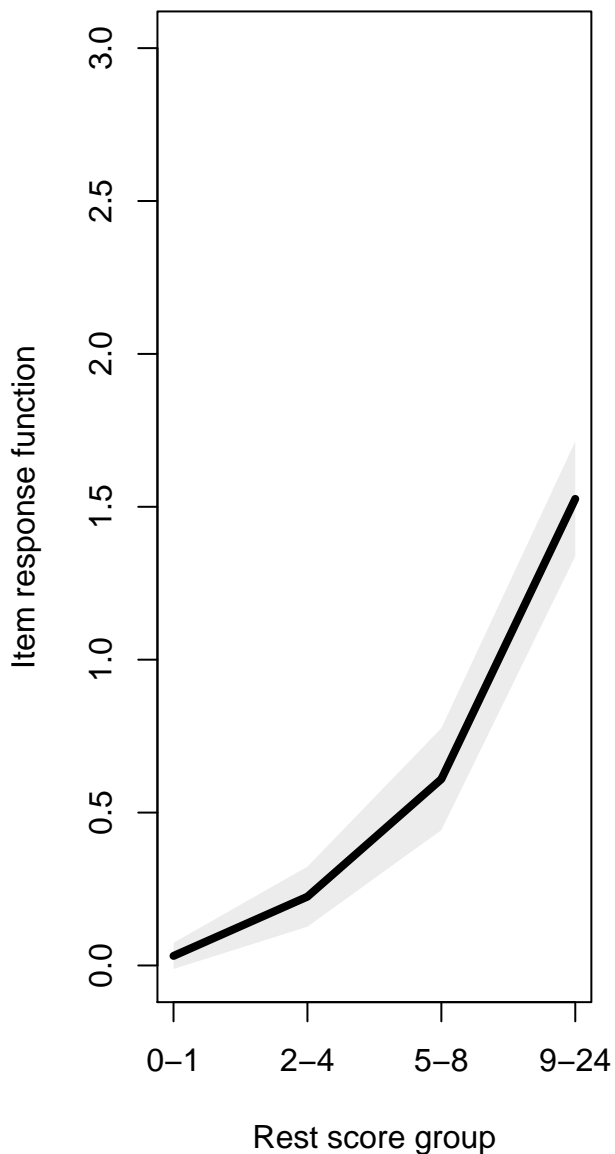

**odi7**

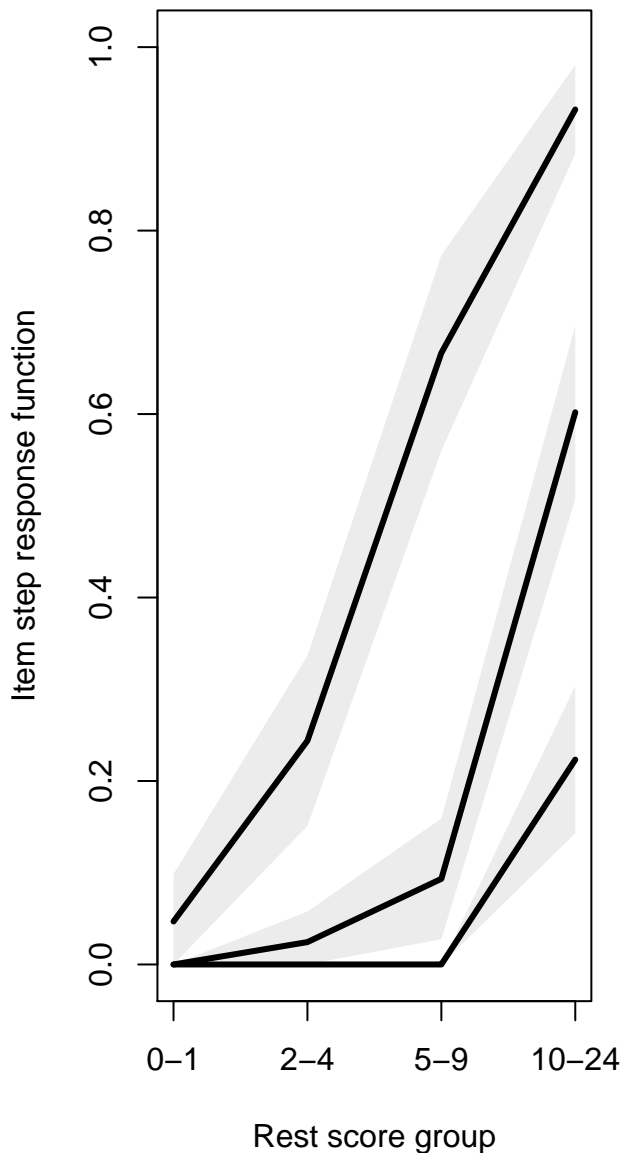

**odi7**

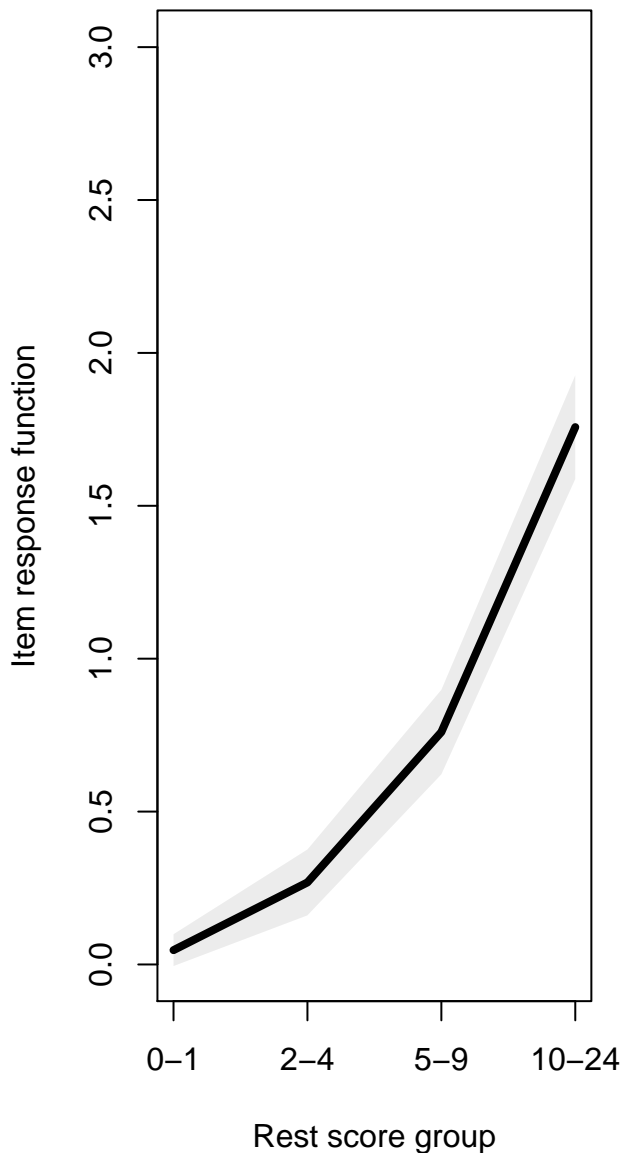

**odi8**

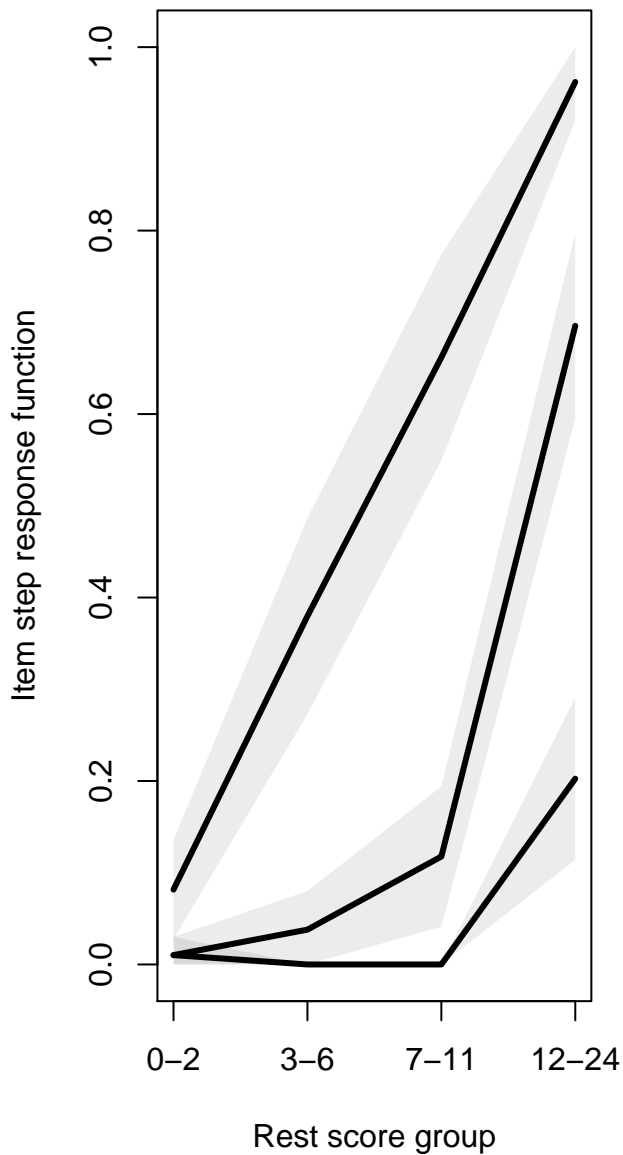

**odi8**

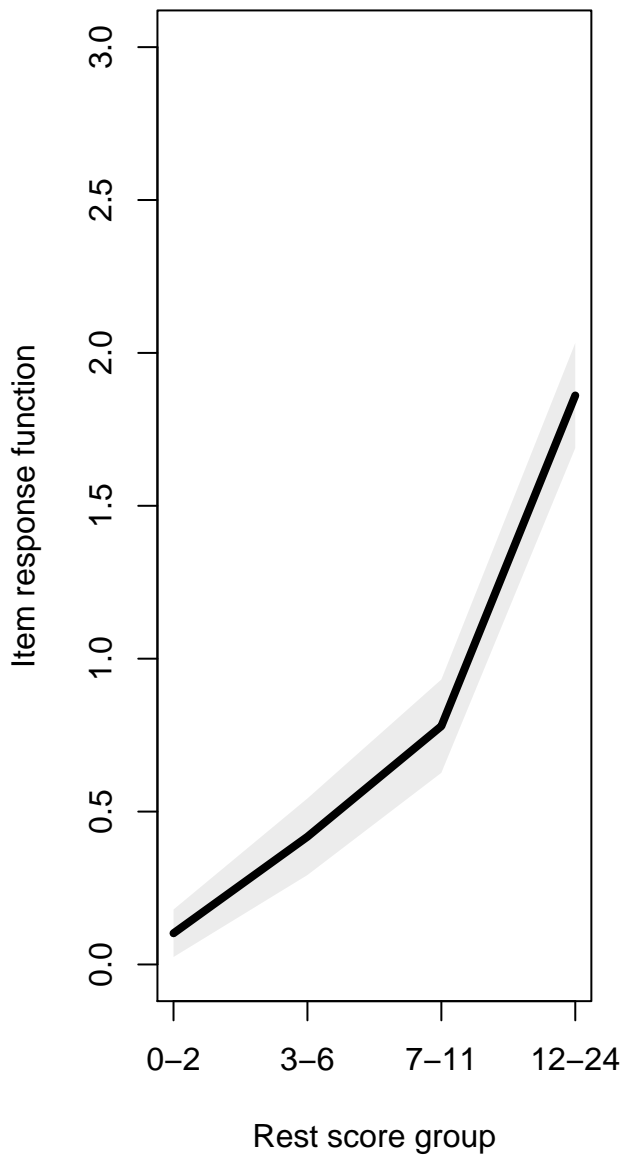

**odi9**

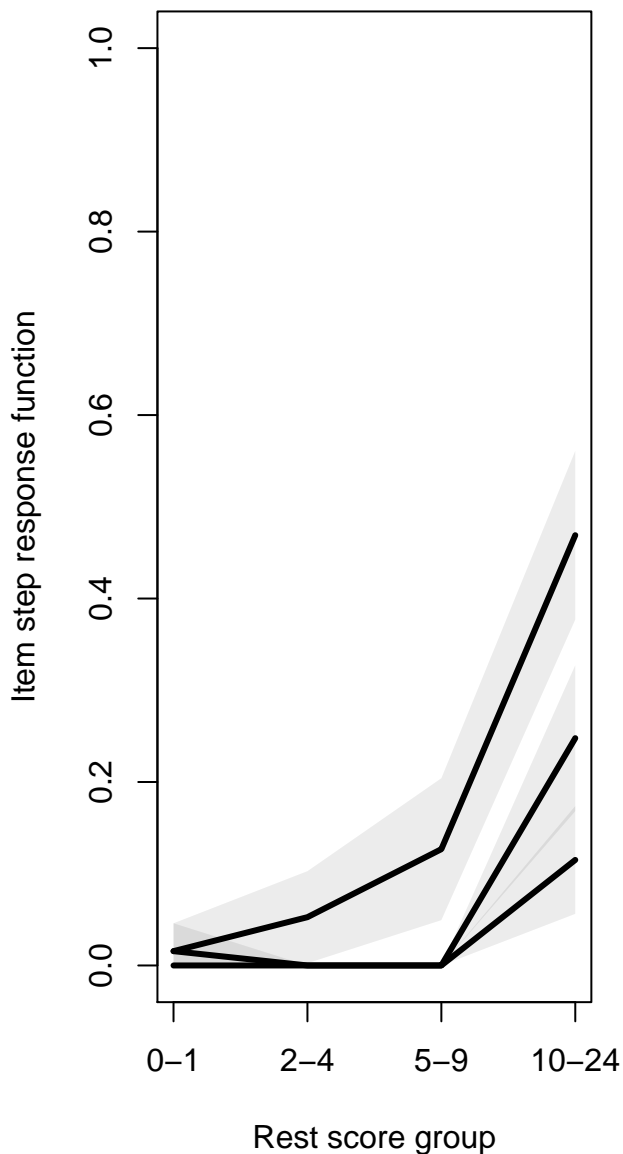

**odi9**

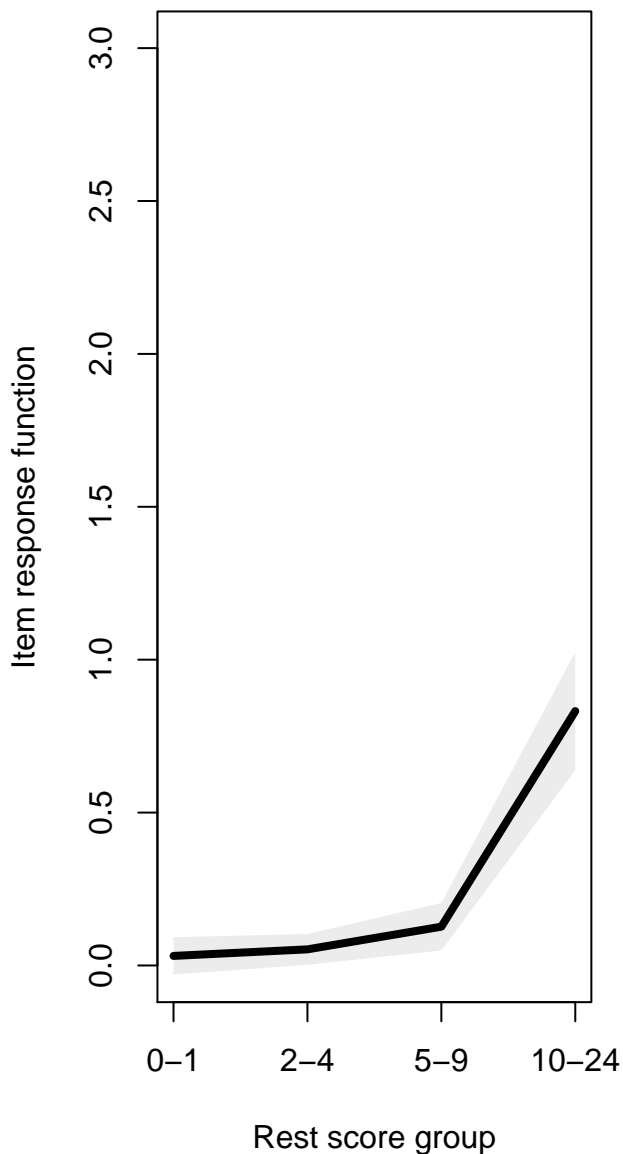

Supplement: S1 File — (PDF) [file pone.0261271.s001.pdf]
