# Supplementary material for: Validation and measurement invariance of the Occupational Depression Inventory in South Africa
Source: PLoS One. 2021 Dec 16;16(12):e0261271. doi: 10.1371/journal.pone.0261271 (PMC8675679; doi:10.1371/journal.pone.0261271)
Supplement: S2 File — (PDF) [file pone.0261271.s002.pdf]

S2 File

Differential item functioning (DIF) – sex

As can be seen from Table 1, item 4 was flagged for DIF. However, the McFadden pseudo-R^2 indices suggested that the DIF effect was negligible. Additionally, the change in beta parameter for model 1 and model 2 was well below 5% in all instances. Figure 1 shows the plots for item 4 and, as can be seen from the figure (bottom-right), the impact is negligible.

Table 1. DIF test based on participants' sex

Item #	M1 vs M2	M1 vs M3	M2 vs M3	R^2 (M1, M2)	R^2 (M1, M3)	R^2 (M2, M3)	Δβ (M1, M2)
1	0.115	0.177	0.323	0.003	0.004	0.001	0.013
2	0.035	0.095	0.608	0.006	0.006	0.000	0.022
3	0.782	0.646	0.372	0.000	0.001	0.001	0.001
4	0.944	0.002	0.000	0.000	0.014	0.014	0.000
5	0.014	0.013	0.107	0.008	0.011	0.003	0.009
6	0.096	0.015	0.017	0.004	0.012	0.008	0.001
7	0.575	0.580	0.378	0.000	0.001	0.001	0.001
8	0.666	0.649	0.410	0.000	0.001	0.001	0.001
9	0.041	0.084	0.372	0.009	0.011	0.002	0.021

Notes. M = model number chi-square test; statistical significance < 0.01; R^2 = McFadden pseudo-R^2; Δβ = change in beta for model 1 and model 2.

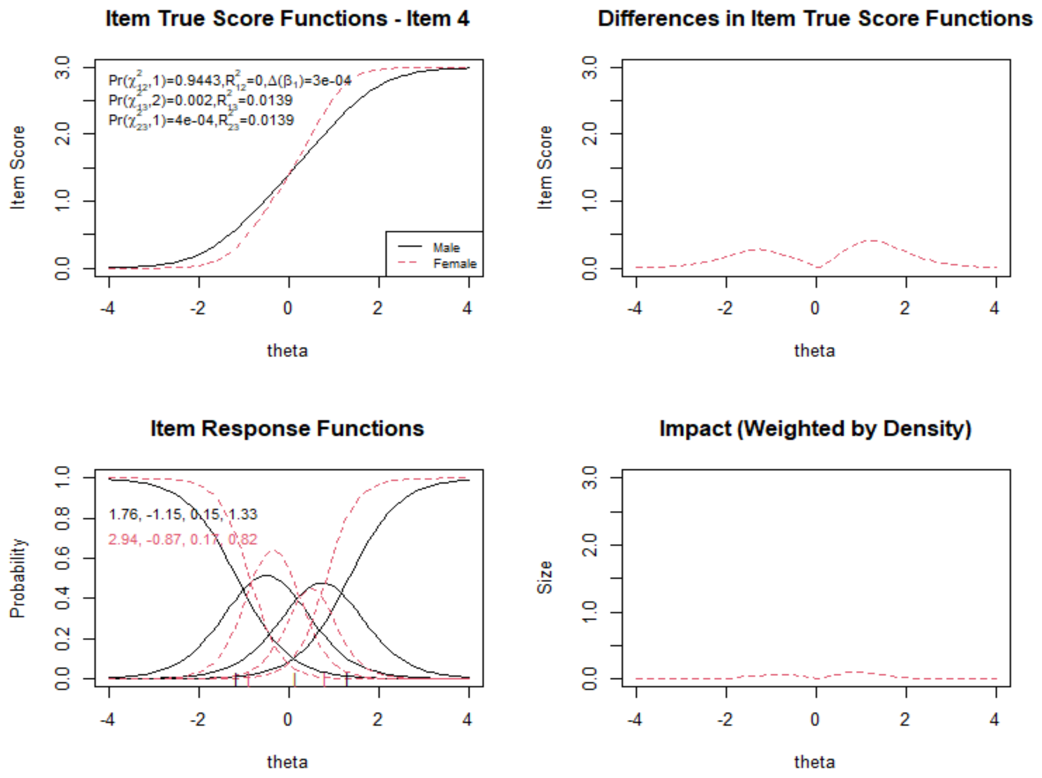

Differential item functioning (DIF) – ethnicity

As can be seen from Table 2, no items were flagged for DIF. Specifically, there were no significant difference tests at the $p < 0.01$ level between models and all changes in beta parameters were well below 5%. Therefore, it was not possible to generate any meaningful plots to accompany the table.

Table 2. DIF test based on participants' ethnicity

Item #	M1 vs M2	M1 vs M3	M2 vs M3	R^2 (M1, M2)	R^2 (M1, M3)	R^2 (M2, M3)	Δβ (M1, M2)
1	0.244	0.438	0.622	0.003	0.005	0.001	0.011
2	0.262	0.494	0.699	0.004	0.004	0.001	0.013
3	0.533	0.611	0.489	0.002	0.003	0.002	0.002
4	0.558	0.165	0.070	0.001	0.008	0.006	0.006
5	0.371	0.557	0.600	0.003	0.004	0.001	0.003
6	0.845	0.199	0.059	0.001	0.009	0.008	0.002
7	0.723	0.572	0.322	0.001	0.004	0.003	0.001
8	0.868	0.865	0.608	0.000	0.002	0.002	0.002
9	0.181	0.133	0.162	0.011	0.022	0.012	0.018

Notes. M = model number chi-square test; statistical significance < 0.01; R^2 = McFadden pseudo-R^2; Δβ = change in beta for model 1 and model 2.

Differential item functioning (DIF) – sample

Table 3 shows that items 1, 3, 4, 8, and 9 were flagged for DIF. For items 1, 3, 4, and 8, the impacts were of negligible magnitude. Item 9's change in beta was 6.6% (i.e., above the 5% cut-off). However, given the intensity of the item, the item text itself, and the difference in sample sizes (327 versus 2254), the impact can be considered minor again.

Table 3. DIF test for the ODI based on participants' sample

Item #	M1 vs M2	M1 vs M3	M2 vs M3	R^2 (M1, M2)	R^2 (M1, M3)	R^2 (M2, M3)	Δβ (M1, M2)
1	0.000	0.000	0.180	0.009	0.003	0.003	0.000
2	0.917	0.937	0.729	0.000	0.000	0.000	0.000
3	0.000	0.000	0.261	0.011	0.008	0.008	0.000
4	0.000	0.000	0.882	0.007	0.005	0.005	0.000
5	0.126	0.301	0.812	0.000	0.000	0.000	0.000
6	0.058	0.051	0.126	0.002	0.001	0.001	0.000
7	0.587	0.281	0.134	0.000	0.000	0.000	0.000
8	0.001	0.001	0.113	0.003	0.002	0.002	0.000
9	0.000	0.000	0.909	0.066	0.033	0.033	0.000

Notes. M = model number chi-square test; statistical significance < 0.01; R^2 = McFadden pseudo-R^2; Δβ = change in beta for model 1 and model 2.

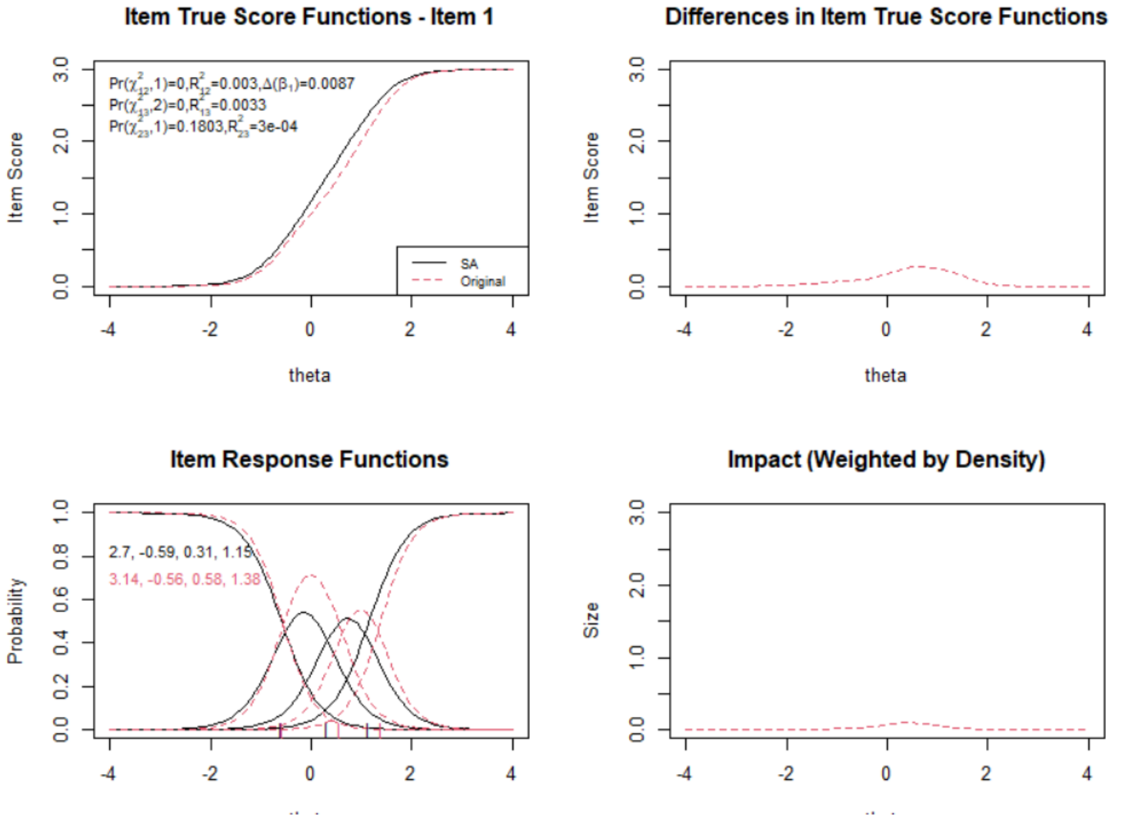
Item True Score Functions - Item 1
Pr(χ²₁₂,1)=0,R²₁₂=0.003,Δ(β₁)=0.0087
Pr(χ²₁₃,2)=0,R²₁₃=0.0033
Pr(χ²₂₃,1)=0.1803,R²₂₃=3e-04
SA
Original
Item Score
theta
Differences in Item True Score Functions
Item Score
theta
Item Response Functions
2.7, -0.59, 0.31, 1.15
3.14, -0.56, 0.58, 1.38
Probability
Impact (Weighted by Density)
Size

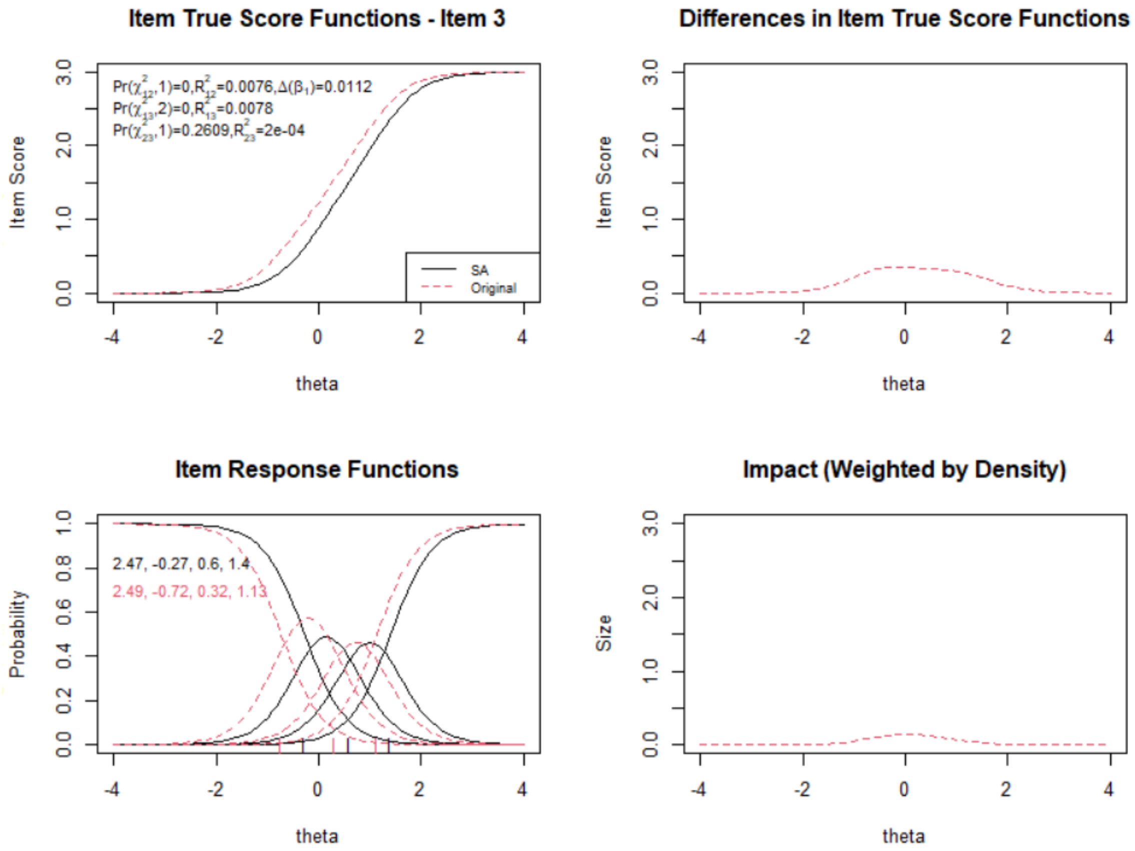
Item True Score Functions - Item 3
Pr(χ²₁₂,1)=0,R²₁₂=0.0076,Δ(β₁)=0.0112
Pr(χ²₁₃,2)=0,R²₁₃=0.0078
Pr(χ²₂₃,1)=0.2609,R²₂₃=2e-04
SA
Original
Item Score
theta
Differences in Item True Score Functions
Item Score
theta
Item Response Functions
2.47, -0.27, 0.6, 1.4
2.49, -0.72, 0.32, 1.13
Probability
theta
Impact (Weighted by Density)
Size
theta

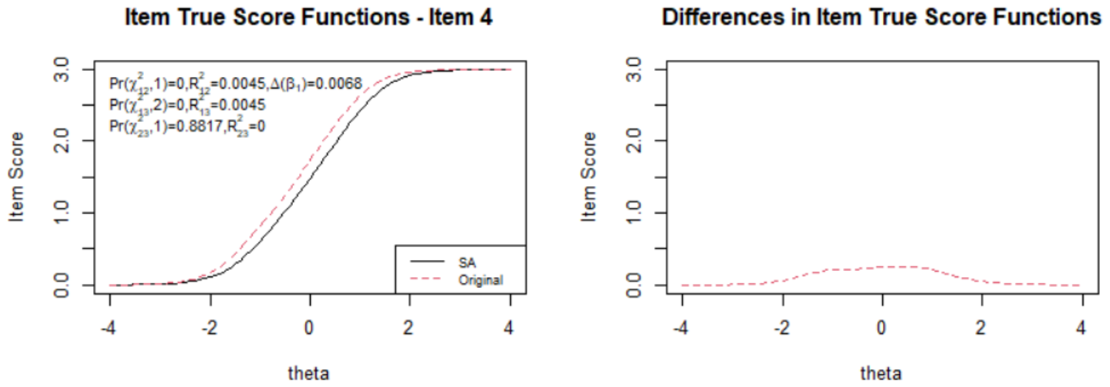
Item True Score Functions - Item 4
Pr(χ²₁₂,1)=0,R²₁₂=0.0045,Δ(β₁)=0.0068
Pr(χ²₁₃,2)=0,R²₁₃=0.0045
Pr(χ²₂₃,1)=0.8817,R²₂₃=0
SA
Original
Item Score
theta
Differences in Item True Score Functions
Item Score
theta
Item Response Functions
2.34, -1.04, 0.08, 0.92
2.57, -1.33, -0.17, 0.68
Probability
theta
Impact (Weighted by Density)
Size
theta

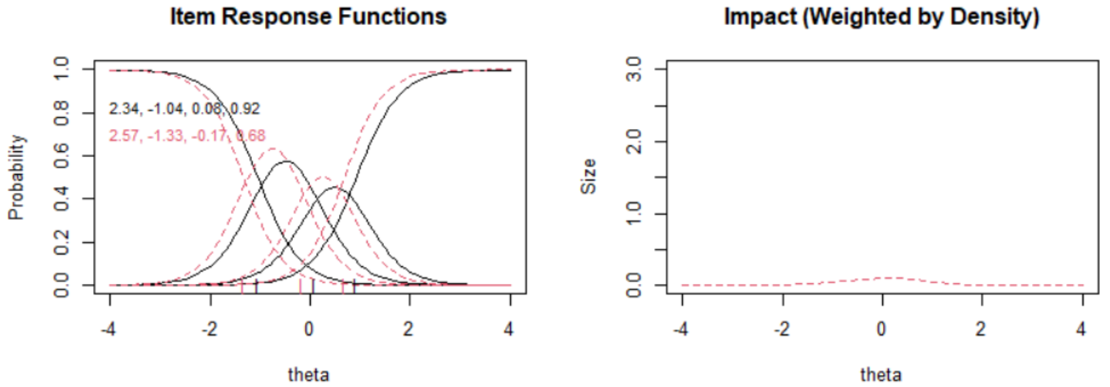
Item True Score Functions - Item 8
Pr(χ²₁₂,1)=5e-04,R²₁₂=0.0019,Δ(β₁)=0.0027
Pr(χ²₁₃,2)=6e-04,R²₁₃=0.0023
Pr(χ²₂₃,1)=0.1133,R²₂₃=4e-04
SA
Original
Item Score
theta
Differences in Item True Score Functions
Item Score
theta
Item Response Functions
3.1, -0.05, 0.88, 1.79
3.21, -0.15, 0.7, 1.49
Probability
theta
Impact (Weighted by Density)
Size
theta

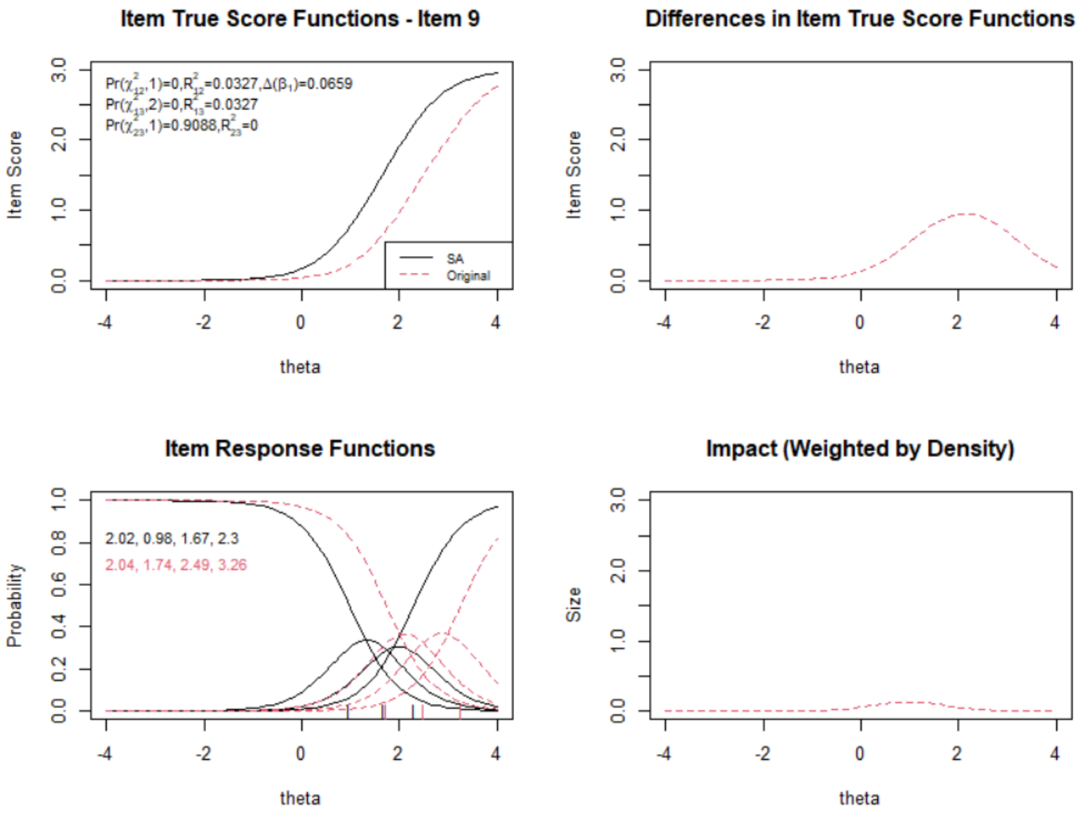

Differential item functioning (DIF) – age group

Only the first three age groups were viable for DIF testing due to group sizes. As can been seen from Table 4, no items were flagged for DIF. None of the model chi-square tests were significant and the changes in beta parameters were all well below 5%.

Table 4. DIF test based on participants' age group

Item #	M1 vs M2	M1 vs M3	M2 vs M3	R^2 (M1, M2)	R^2 (M1, M3)	R^2 (M2, M3)	$\Delta\beta$ (M1, M2)
1	0.131	0.183	0.341	0.010	0.005	0.008	0.003
2	0.866	0.509	0.222	0.000	0.000	0.005	0.004
3	0.488	0.491	0.372	0.006	0.002	0.005	0.003
4	0.840	0.661	0.357	0.005	0.001	0.003	0.003
5	0.648	0.875	0.840	0.001	0.001	0.002	0.001
6	0.279	0.612	0.935	0.011	0.004	0.004	0.000
7	0.422	0.152	0.083	0.005	0.003	0.010	0.008
8	0.421	0.736	0.872	0.018	0.003	0.004	0.001
9	0.599	0.185	0.075	0.018	0.004	0.022	0.018

Notes. M = model number chi-square test; statistical significance < 0.01; R^2 = McFadden pseudo-R^2; $\Delta\beta$ = change in beta for model 1 and model 2.
